# Benefit and Preference of Propranolol Over Metoprolol in Thyrotoxicosis-Induced Atrial Fibrillation: A Case Report and Review of Literature

**DOI:** 10.7759/cureus.34474

**Published:** 2023-01-31

**Authors:** Mukosolu F Obi, Vikhyath Namireddy, Yash Garg, Manjari Sharma

**Affiliations:** 1 Internal Medicine, Wyckoff Heights Medical Center, New York, USA; 2 Medicine, St. George's University School of Medicine, True Blue, GRD; 3 Internal Medicine, Wyckoff Heights Medical Center, Brooklyn, USA

**Keywords:** standard of care, hyperthyroidism, atrial fibrillation/flutter, amiodarone induced thyrotoxicosis, beta-blockers

## Abstract

Atrial fibrillation is a common manifestation seen in patients with hyperthyroidism and thyroid storm. The presence of excess thyroid hormone (TH) alters adrenergic receptors in the heart and blood vessels, thereby causing an increase in sympathetic function and atrial fibrillation as a sequela of this excess circulating hormone. Excess thyroid hormone (T3) shortens the action potential of cardiomyocytes in the pulmonary vein, which facilitates the generation of reentrant circuits causing atrial fibrillation. Thyroid hormone can regulate cardiac beta-adrenergic receptor expression leading to enhanced catecholamine sensitivity of beta-adrenergic coupled cardiac response. We present a case of a 64-year-old female with a history of hypertension (HTN), nonobstructive coronary artery disease (CAD), congestive heart failure (CHF) [ejection fraction (EF) 35-40%], chronic obstructive pulmonary disease (COPD) on long-term oxygen therapy (LTO2), obstructive sleep apnea (OSA)/hypoventilation syndrome, atrial flutter/atrial fibrillation with a loop recorder on rivaroxaban, and obesity who presented to the emergency department (ED) with gastroenteritis symptoms precipitating difficulty breathing and atrial fibrillation with a rapid ventricular response (HR 140-150) requiring ICU admission for rate and rhythm control. During the course of hospitalization, she was treated with an amiodarone infusion, which induced thyrotoxicosis and increased the ectopic electrical activity in the atrium, worsening atrial fibrillation. On day 3, amiodarone was stopped, and IV esmolol and metoprolol tartrate PO were continued with no resolution of atrial fibrillation. The patient was transitioned to propranolol, which achieved adequate heart rate control prior to discharge. The aim of our review is to highlight that propranolol should be used over metoprolol in patients with hyperthyroidism-induced atrial fibrillation due to the effect of propranolol on blocking the activity of T4 conversion to active T3 and, as such, blocking its effect on cardiac myocytes, terminating reentrant atrial excitation.

## Introduction

Atrial fibrillation is the most common cardiac arrhythmia seen in the state of hyperthyroidism in 0-15% of patients [1]. It is the abnormal or irregular heart rhythm that causes dysfunction in the upper chambers of the heart. Electrical impulses generated by atrial fibrillation cause inadequate pumping of blood from the upper chambers (atria) of the heart. This abnormal filling leads to the formation of blood clots, thereby predisposing the patient to strokes [2]. Risk factors such as aging, obstructive sleep apnea, diabetes, hypertension, obesity, thyroid disease, renal dysfunction, COVID-19, and dyslipidemia may act synergistically to cause atrial fibrillation [1]. The myocardial and vascular endothelial tissues have receptors for thyroid hormones (THs) and are sensitive to changes in the concentrations of circulating thyroid hormones. Hyperthyroidism increases the arrhythmogenic activity of cardiomyocytes in the pulmonary veins. Thyroid-stimulating hormone (TSH) levels of ≤0.1 mIU/L have been linked to a threefold increase in atrial fibrillation risk [3]. The effect of thyroid hormones on the cardiomyocytes via genomic and nongenomic actions has been well documented [3]. Cardiac excitation is a result of the cardiac action potential, which is caused by electrolytes like potassium, sodium, and calcium. These electrolytes have specific phases in the cardiac action potential cycle where their increased concentration enables excitation or dormancy of the cardiomyocyte. Increasing the transcription and upregulation of potassium, sodium, calcium channel subunits, and gap junctions in the hyperthyroid state exaggerates catecholamine tone, altering cell-cell coupling and resulting in cardiomyocyte excitation. The non-genomic effect involves an independent signalling mechanism. In the hyperthyroid state, upregulation of electrogenic proteins includes voltage-gated potassium channels, Na^+^/K^+^ ATPase, and Na^+^/Ca^2+^ ATPase activities, which influence cardiac excitability [4]. The cornerstone of medical therapy in a hyperthyroid state is to decrease the levels of circulating T3 in the blood and to inhibit the TH peripheral effect through the β-adrenergic blockade. Propranolol is the preferred agent for β-adrenergic blockade due to its additional effect of blocking the peripheral conversion of inactive thyroxine (T4) to active form T3 via inhibition of the 5'-monodeiodinase that converts T4 to T3 [5] compared to metoprolol. This case report with a literature review provides current data on the preference of propranolol over metoprolol in patients with hyperthyroidism and serves as a cautionary note in patients with contraindications.

## Case presentation

**Figure 1:**
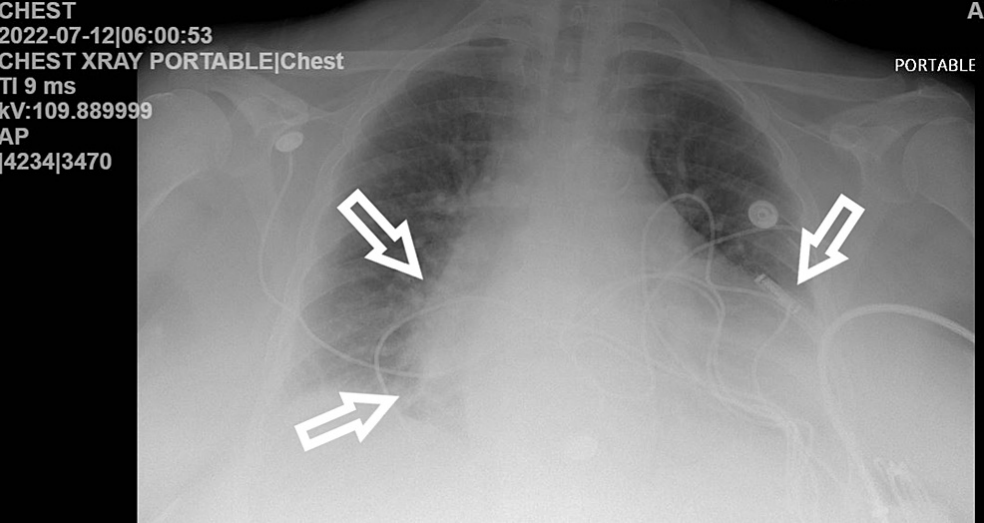
Chest X-ray: consistent with cardiomegaly with hazy perihilar and lower lobe opacities.

**Figure 2:**
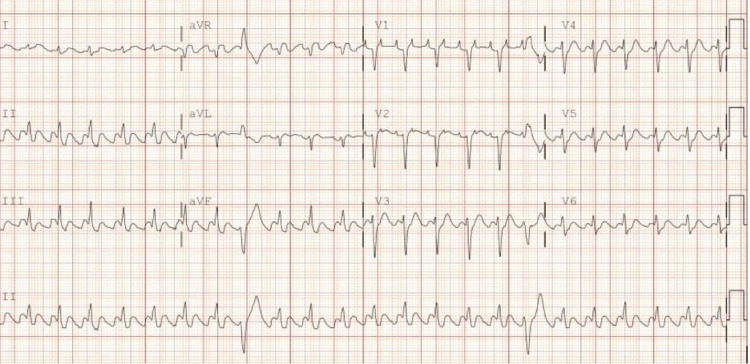
EKG on admission showed atrial flutter with 2:1 block.

We present a 64-year-old female with a past medical history of arterial hypertension, nonobstructive coronary artery disease, chronic heart failure (EF 35-40%), chronic obstructive pulmonary disease (COPD) on long-term oxygen therapy (LTO2), obstructive sleep apnea (OSA)/hypoventilation syndrome, atrial flutter/atrial fibrillation with a loop recorder on rivaroxaban, morbid obesity, and chronic venous stasis who presented to the emergency department with the chief complaints of nausea, vomiting, and diarrhea associated with shortness of breath. The patient reported family members with similar symptoms (gastroenteritis) for the past four days. Upon presentation, the patient was hypothermic with a temperature of 96.1 °F in triage, with laboured breathing and pursed lips. EMS reported that the patient tried continuous positive airway pressure (CPAP) at home without resolution of symptoms. The patient denied having chest pain and coughing but reported heart palpitations. The patient’s vitals were reported as HR: 200 BPM, respiratory rate: 50 BPM, blood pressure: 180/114 mmHg, with a significant increase in work of breathing. The patient was placed on bilevel positive airway pressure (BIPAP) with a resolution of tachypnea, however, the heart rate remained elevated. The chest X-ray (CXR) demonstrated cardiomegaly with hazy perihilar and lower lobe opacities and questionable small pleural effusions. Findings were consistent with interstitial edema/fluid overload, with the possibility of infection not excluded (Figure 1). EKG on admission prior to amiodarone infusion was significant for atrial flutter with 2:1 block (Figure 2). The patient received Diltiazem 20 mg and 35 mg IV push (IVP) with no improvement. Metoprolol tartrate 5 mg IV push was given but despite the intervention, the patient remained with atrial flutter/atrial fibrillation rapid ventricular response (RVR) with HR 140-150 BPM. Cardiology was consulted, and the patient was given both an amiodarone bolus and an infusion before being transferred to the ICU for closer monitoring. The recommendation from the cardiologist was to resume home medication, which was metoprolol tartrate 100 mg every 12 hours in addition to the infusion. Overnight, the patient's HR was 180-220 BPM and BP was 179/96 mmHg, with new complaints of chest tightness, progressive shortness of breath, and unchanged mentation. A repeat CXR showed acute pulmonary edema. Cardiology was re-consulted with the following recommendations: start IV 40 mg and 20 mg of Furosemide with an additional bolus of 150 mg of amiodarone and start esmolol and nitroglycerin infusion. The patient’s HR reduced to 140 bpm, but the rhythm still remained in atrial fibrillation with RVR. High-sensitivity troponin was reported at 996.4-1094 ng/L likely due to demand ischemia in the setting of tachyarrhythmia. The patient was also treated for sepsis secondary to community-acquired pneuma with IV antibiotics.

On day 2, the thyroid panel resulted in an indication of hyperthyroidism. Patient with no prior history of thyroid disease and not on any thyroid medication showed low TSH and elevated T4 after amiodarone infusion (Table 1).

**Table 1:** Morning lab on day 2 indicative of hyperthyroidism with TSH <0.005, FT4 2.18.

Haematology		Chemistry	
WBC	↑↑23.90	Calcium level	9.9
Haemoglobin	↑↑16.3	Albumin level	↓↓3.1
Haematocrit	↑↑51.5	Total protein	6.7
MCV	97.1	Sodium level	144
MCH	30.8	Potassium level	4.1
MCHC	31.7	Chloride level	106
RDW	12.6	CO_2_ level	28
Platelet count	271	Blood urea nitrogen	↑↑22
MPV	8.6	Creatinine	0.69
Neutrophils	↑↑86.7	Glucose	↑↑149
Lymphocytes	↓↓4.1	Alkaline phosphatase	75
Monocytes	8.9	Sgot-Ast	23
EOS count	↓↓0.2	Sgpt-Alt	18
Basophils	0.1	Total bilirubin	0.6
Nucleated RBC	0	GFR estimation	>60
		Phosphorus, serum	3
		Magnesium, serum	2
		HGB A1C	↑↑6.3
		TSH	↓↓<0.005
		Free T4	↑↑2.18
		Procalcitonin	↑↑2.48
		TPO Ab	1
		TSI Ab	<89
Time interval (troponin)			
	Time 0	Q4	Q8
High sensitivity troponin	↑↑996.4	↑↑1094	↑↑661

**Figure 3:**
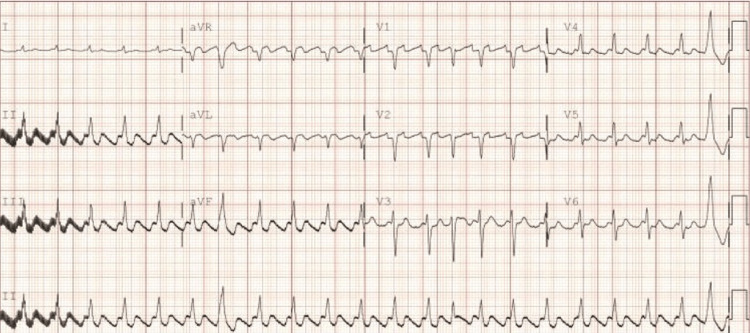
EKG repeat after amiodarone infusion and esmolol - shows persistent AFL with HR 129 bpm.

Upon discovery, amiodarone infusion was discontinued, and an endocrinologist was consulted with the recommendation of starting methimazole at 10 mg every 12 hours for one week and then 10 mg QD with a repeat TSH/T4/T3 in three to four days and also a thyroid peroxidase antibody and thyroid stimulating immunoglobin. A short course of methylprednisolone, 40 mg Q8, was given and tapered. The cardiologist recommended Digoxin 250 mcg IVP followed by a six-hourly repeat dose when telemetry showed HR 130-140 s while still on esmolol continuous infusion. A repeat EKG was significant for atrial fibrillation with RVR (Figure 3).

On day 4 in the ICU, the HR on the telemonitor was 120 bpm, and the patient was transitioned to the regular floor for closer monitoring. The esmolol infusion was discontinued, and the patient was transitioned to Digoxin 125 mcg PO and metoprolol tartrate 50 mg q8. On day 5, HR was 125-130 bpm on tele; medication was adjusted, and repeat TSH was still <0.005 and T4 was 1.19 ng/dL. The patient was placed on metoprolol succinate 200 mg PO QD, and digoxin was discontinued to limit toxicity. The heart rate was still not controlled. At that time, the patient was being titrated down on prednisone, and methimazole was discontinued. Management was changed; propranolol 30 mg q8 was started and later titrated up to 80 mg q8 with significant improvement in HR (103-120 bpm), at which point repeat TSH showed 0.193 (Table 2). The patient was scheduled to follow up outpatient with cardiology one week later for medication transition to metoprolol tartrate 100 mg PO q 12 given the patient's co-morbidities of chronic obstructive lung disease. During the hospital course, it was noted that the patient was on amiodarone at home but later transitioned to metoprolol tartrate when the heart rate was not controlled. Delayed presentation of amiodarone-induced thyroiditis/thyrotoxicosis (AIT) has been documented, and as such, high clinical suspicion is warranted regardless of amiodarone treatment duration [6].

**Table 2:** Lab result with a significant increase in TSH level while the patient is on propranolol.

Chemistry	
Calcium level	9.4
Albumin level.	↓↓3.2
Total protein	↓↓6.2
Sodium level	142
Potassium level	↑↑5.4
Chloride level	102
CO_2_ level	↑↑37
Blood urea nitrogen	↑↑83
Creatinine	1.03
Glucose.	↑↑123
Alkaline phosphatase	50
Sgot-Ast	20
Sgpt-Alt	30
Total bilirubin	0.5
GFR estimation	54
Phosphorus, serum	3
Magnesium, serum	2.3
Thyroid-stimulating hormone	↓↓0.193
Free T4	1.12

## Discussion

Propranolol is a non-selective beta-1 and beta-2 blocker, utilized in states of hyperthyroidism and thyroid storm preferentially due to its effect of blocking the peripheral conversion of inactive T4 to active form T3 [5]. In a hyperthyroid state, extreme amounts of T4 (90% of the circulating thyroid hormone) are released from the thyroid follicles and then converted to T3 (a more potent hormone) by two enzymes: monodeiodinase type I and monoiodinase type II. T3 is lipophilic and transported by thyroid-binding proteins. Carrier protein (TBG - thyroid binding globulin) allows an easy transition into target cells where it interacts with genes, increasing the expression of proteins like Na/K ATPase [7,8]. The thyroid hormone exerts its cardiovascular effects by increasing the gene transcription of cardiac myocyte proteins. Thyroid hormones upregulate sarcoplasmic calcium ATPase, myosin heavy chain alfa, voltage-gated K^+^ channels, Na^+^ channels, and beta1 adrenergic receptors [7,9]. Beta (β) adrenergic receptors are part of the principle stimulating pathways of the sympathetic nervous system. These G-coupled ligand receptors are activated predominantly by catecholamines. An increase in β-1 adrenergic receptors and their sensitivity to catecholamines (norepinephrine and epinephrine) leads to an increase in heart rate, and contractility with a combined effect of an increase in cardiac output/blood pressure. Beta-1 adrenergic receptors are predominant in the SA and atrioventricular nodes, as the thyroid hormone increases its sensitivity to catecholamines and shortens the action potential duration (APD), resulting in atrial fibrillation [7,10]. Documented studies using rabbit pulmonary vein cardiomyocytes reported that thyroid hormones decrease the action potential duration in pulmonary vein cardiomyocytes, resulting in the facilitation of reentrant circuits [7]. Propranolol blocks the activity of monodeiodinase type I, which decreases peripheral conversion of T4 to T3. Propranolol increases the serum reverse T3 concentration with lesser changes in other serum thyroid hormone levels compared to metoprolol [11,12].

The circulating thyroid hormone decreases systemic vascular resistance and, as such, increases cardiac output and decreases peripheral vascular resistance, leading to high-output heart failure. Propranolol with beta-2 blockade increases systemic vascular resistance, abating cardiovascular collapse [1,13]. In high-output HF, the elevation in cardiac output is greater than that required to meet metabolic demand. Defined by an increase in cardiac output, low systemic vascular resistance due to peripheral vasodilation, and a difference in low arterial-venous oxygen content due to increased oxygen consumption as a result of increased metabolic demand. The ineffective blood volume and pressure lead to the body's activation of the sympathetic nervous system (further precipitating tachycardia), activation of the renin-angiotensin-aldosterone axis, and increased serum vasopressin (to maintain vascular tone). Excess thyroid hormones can surpass the compensatory capacity of coronary vasodilation, precipitating ischemia and systolic dysfunction [14]. With the use of propranolol both the effect of excess thyroid hormones and the body's mechanism to compensate for the cardiovascular collapse will be atoned [15]. Propranolol is highly lipid soluble, allowing it to become sufficiently concentrated in targeted tissues in order to inhibit the activities of monodeiodinase type I and monoiodinase type II as compared to metoprolol. Propranolol's effect on the reduction of T4's metabolism through the inhibition of monodeiodinase type I can result in the T4 being shunted through the enzyme monodeiodinase type III (5'-D-III), resulting in the production of 3,3',5'-triiodothyronine (reverse T3 or rT3), which is metabolically inactive [16].

Noncardioselective β-blockers (Propranolol) in both thyrotoxic crisis and uncomplicated hyperthyroidism have been known to be the standard of therapy [5,17]. However, there should be caution with usage, especially in patients with pre-established systolic heart failure dysfunction and chronic obstructive pulmonary disease. The effect of propranolol is slow, occurring over 7-10 days. Propranolol in a high dose slowly decreases serum triiodothyronine (T3) concentrations by as much as 30% [18]. Caution should be exercised in the use of propranolol infusion as its slow onset could lead to a drop in mean arterial pressure, causing cardiovascular collapse, especially in patients with decreased left ventricular function, requiring an ICU setting for close monitoring and the utilization of inotropic agents and vasopressors if required. For patients in hyperthyroid state/thyrotoxicosis without heart failure, beta-blockade brings symptomatic relief without cardiac output (CO) compromise. On the contrary, in patients with hyperthyroid/thyrotoxicosis and low-output cardiac failure, the compensatory role of maintaining CO is due to a thyroid-induced hyperadrenergic state, thereby leading to hemodynamic instability and a significant fall in CO because of the administration of beta-blockade halting this compensatory mechanism [5,8]. This is supported by some published case reports [19,20]. The main reason caution should be exercised when using beta-blockade is because of the exaggerated sensitivity of the beta-adrenergic receptors caused by excess thyroid hormones. Our patient had a past medical history of chronic systolic heart failure with an ejection fraction of 35-40% and presented with flash pulmonary edoema and difficulty breathing. So, IV propranolol was not considered when it was known that the patient's persistent atrial fibrillation with RVR with the intermittent transition to atrial flutter was due to amiodarone infusion causing precipitating hyperthyroid state. Thus, esmolol was initiated in the ICU setting for closer monitoring, and quick instructions were given to use vasopressor and inotropic support in the event of cardiogenic shock. Our patient already has a history of atrial fibrillation and was on metoprolol at home prior to the event that precipitated her symptoms. Our patient had all the risk factors for developing atrial fibrillation, like diabetes, uncontrolled hypertension, morbid obesity, and obstructive sleep apnea. Thus, the temporal use of propranolol was warranted to abate hyperthyroidism worsening atrial fibrillation.

## Conclusions

The established cornerstones in medical therapy of patients in hyperthyroid states include decreasing circulating levels of T3 in the bloodstream and the inhibition of the hormone’s peripheral effects through β-adrenergic blockade. The sensitivity of circulating catecholamines is exaggerated in hyperthyroid states by the hormone’s ability to increase β-adrenergic receptor density through the amplified formation and reduced degradation. In an attempt to impede this hyperadrenergic state, non-cardio-selective β-blockers (NCBB) have been used. Due to its additional effect of blocking the peripheral conversion of inactive T4 to active T3, propranolol has been the preferred NCBB. Although propranolol is preferred and beneficial in patients in a hyperthyroid state, caution should be exercised and comorbid contradictions investigated prior to use. This case report concisely emphasized significant symptom improvement in our patient when propranolol was initiated in the treatment regimen, although her overall outcome might be synergistic due to the fact that the patient was on metoprolol at home prior to amiodarone-induced hyperthyroidism, and as such, upregulation of beta-1 adrenergic receptor and sensitivity tapered and might have contributed to the patient's hemodynamic stability once esmolol infusion was initiated. Follow-up management for our patient was to transition back to her home dose of metoprolol after two weeks of propranolol and an electrophysiology evaluation for catheter ablation around the pulmonary veins to isolate the trigger that initiates and maintains her long-term persistent atrial fibrillation.
